# Giant Kerr nonlinearity and low-power gigahertz solitons via plasmon-induced transparency

**DOI:** 10.1038/srep13780

**Published:** 2015-09-08

**Authors:** Zhengyang Bai, Guoxiang Huang, Lixiang Liu, Shuang Zhang

**Affiliations:** 1 State Key Laboratory of Precision Spectroscopy and Department of Physics, East China Normal University, Shanghai 200062, China; 2 School of Physics and Astronomy, University of Birmingham, Birmingham B15 2TT, UK

## Abstract

We propose a method to enhance Kerr nonlinearity and realize low-power gigahertz solitons via plasmon-induced transparency (PIT) in a new type of metamaterial, which is constructed by an array of unit cell consisting of a cut-wire and a pair of varactor-loaded split-ring resonators. We show that the PIT in such metamaterial can not only mimic the electromagnetically induced transparency in coherent three-level atomic systems, but also exhibit a crossover from PIT to Autler-Townes splitting. We further show that the system suggested here also possess a giant third-order nonlinear susceptibility and may be used to create solitons with extremely low generation power. Our study raises the possibility for obtaining strong nonlinear effect of gigahertz radiation at very low intensity based on room-temperature metamaterials.

Electromagnetically induced transparency (EIT) is a typical quantum interference effect firstly discovered in coherent three-level atomic systems. In recent years, atomic EIT has found many interesting and important applications from precision laser spectroscopy to quantum information^1^. Recently, many efforts have been made on the classical analogue of EIT in various systems, such as coupled resonators^2^ and electric circuits^3^. Especially, the plasmonic analogue of EIT in metamaterials, called plasmon-induced transparency (PIT)^4^,^5^, has attracted growing interest^6^,^7^,^8^,^9^,^10^. PIT metamaterials can not only work in different regions of radiation frequency but also be used to design chip-scale optical devices. Many applications of PIT metamaterials have been proposed, including optical buffers, highly sensitive sensors, ultrafast optical switches, and light storage, etc^11^,^12^,^13^,^14^.

However, most studies on PIT metamaterials reported up to now are mainly focused on linear propagation regime. Because many applications of PIT require a long-distance propagation, a robust transmission of radiation pulses is needed. Due to the highly resonant (and hence dispersive) character inherent in PIT systems, a linear pulse usually undergoes a significant deformation during propagation. Thus it is necessary to extend the PIT to nonlinear propagation regime.

Here we propose a scheme to enhance Kerr nonlinearity and realize low-power gigahertz solitons via PIT. We suggest a new type of metamaterial and show that the system can not only mimic the EIT of three-level atomic systems, but also display a crossover to Autler-Townes splitting (ATS)^15^ (i.e. *PIT-ATS crossover*) when the bright and dark modes of the system is changed from weak to strong coupling regions. We further show that the system can possess giant third-order nonlinear optical susceptibility and soliton pulses with very low generation power can be produced. Our work raises the possibility for obtaining strong nonlinear effect for gigahertz radiation at very low intensity based on room-temperature metamaterials.

Before preceding, we note that nonlinear metamaterials were considered in refs 16,17 where nonlinearity is realized by embedding a nonlinear medium into split-ring resonator (SRR) slits, and in refs 18, 19, 20 where nonlinearity is obtained by inserting nonlinear elements into SRR slits. Furthermore, nonlinear localized structures in metamaterials were also suggested in refs 20, 21, 22. However, our work is different from refs 16, 17, 18, 19, 20, 21, 22. First, no PIT was used in refs 16, 17, 18, 19, 20, 21, 22 (see also the recent review^23^). Second, the giant Kerr nonlinearity obtained in our system is very large due to the PIT effect. Third, the solitons discovered here are very different from those obtained before because they have extremely low generation power. We note that recently the authors of refs 24,25 reported the investigation on some nonlinear effects in PIT systems but only a single meta-atom (i.e. not an effective medium) was considered and no Kerr effect and soliton were discussed.

## Results

### Model

**Figure 1:**
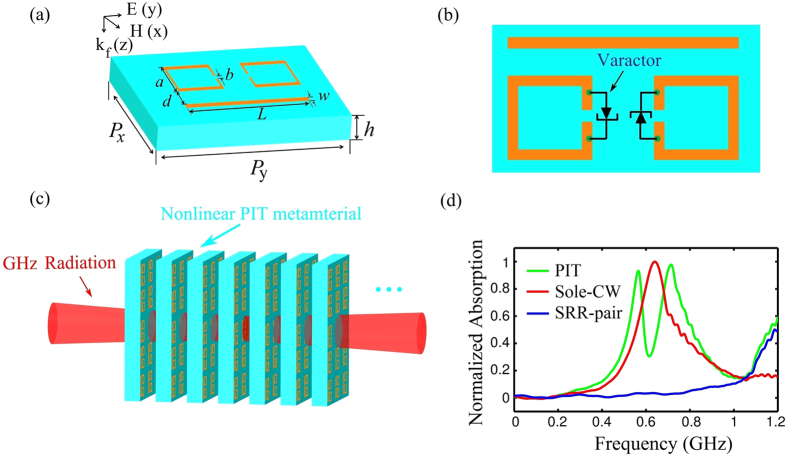
Model and linear dispersion relation and normalized absorption spectrum. (**a**) Schematic of the unit cell of the plasmonic metamaterial, which consists of a CW and a pair of SRRs. Geometrical parameters of the unit cell are *L* = 1.7, *w* = 0.1, *a* = 0.58, *b* = 0.1, *P*_*x*_ = 1.6, *P*_*y*_ = 2.4 (in unit mm). 10-*μ*m-thick aluminium that forms the CW and the SRR-pair pattern is etched on a Si-on-sapphire wafer comprised of 100-*μ*m-thick undoped Si film and 2.1-mm-thick sapphire substrate (i.e. *h* = 2.21 mm). (**b**) SRR pair with a hyperabrupt tuning varactor mounted onto the slits. (**c**) Possible experimental arrangement for the measurement of GHz radiation in the nonlinear PIT metamaterial. (**d**) Normalized absorption spectrum of the CW (red), the SRR-pair (blue) and the unit cell of PIT metamaterial (green).

The metamaterial structure suggested here is an array of unit cell (meta-atom) consisting of a cut-wire (CW) and two SRRs with a nonlinear varactor inserted into their slits (Fig. 1(a,b)). The geometrical parameters of the unit cell are *L* = 1.7, *w* = 0.1, *a* = 0.58, *b* = 0.1, *P*_*x*_ = 1.6, and *P*_*y*_ = 2.4 (in unit mm). 10-*μ*m-thick aluminium that forms the CW and the SRR-pair pattern is etched on a Si-on-sapphire wafer comprised of 100-*μ*m-thick undoped Si film and 2.1-mm-thick sapphire substrate (i.e. *h* = 2.21 mm).

We assume an incident microwave field is collimated on the array with the electric field parallel to the CW. In order to form gigahertz solitons we assume also there are many layers of the unit cell array (Fig. 1(c)). Normalized absorption spectrum of the sole-CW (red), SRR-pair (blue) and the unit cell of PIT metamaterial (green) are shown in Fig. 1(d). The CW array shows a typical localized surface plasmon resonance, while the SRRs support an inductive-capacitive (LC) resonance at the same frequency. The CW is directly excited by the incident electric field along the CW, while the SRRs are weakly coupled to the incident field due to the perpendicular orientation of the field.

Similar to the case in ref. 7, the CW in the unit cell may function as an optical dipole antenna and thus can serve as a bright mode. The resonance frequency of the CW can be tuned by varying its spatial dimension. The two SRRs in the unit cell may function as dark modes, which may be symmetric or antisymmetric. The antisymmetric mode has counter-propagating currents on the two SRRs; therefore, there is no direct electrical dipole coupling with the radiation wave, and it can be considered as a dark mode with a significantly longer dephasing time. The resonance frequency of this dark mode is designed to coincide with that of the bright mode. Hence the CW and the SRRs serve respectively as the bright and dark modes in our excitation scheme, which leads to a dip at the center of the broad peak for the absorption spectrum (i.e. PIT).

**Figure 2:**
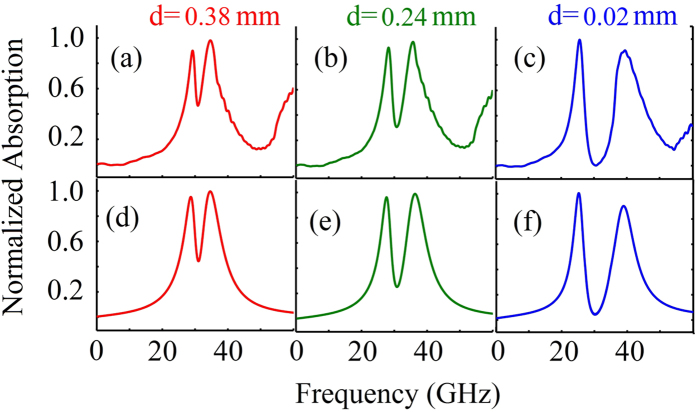
Absorption spectrum. Numerical results of the normalized absorption spectrum of the metamaterial (Fig. 1) for (**a**) *d* = 0.38, (**b**) *d* = 0.24, and (**c**) *d* = 0.02 (in unit mm), respectively. Analytical results given in (**d**–**f**) are obtained from solving the model Eqs (1) (2) and (3) in linear regime.

In panels (a), (b), (c) of Fig. 2 we show respectively the numerical result of normalized absorption spectrum of the system versus frequency for separation *d* = 0.38, 0.24, and 0.02 (in unit mm), obtained by using the commercial finite difference time domain soft-ware package (CST Microwave Studio). We observe that a PIT transparency window in the absorption spectrum opens; furthermore, the transparency window becomes wider and deeper as *d* is reduced. These phenomena are clear indications of PIT-ATS crossover and will be analyzed in detail below.

The dynamics of the bright and dark modes in the unit cell at the position **r** = (*x*, *y*, *z*) can be described by the coupled Lorentz oscillator model^4^,^7^


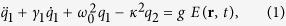



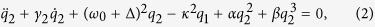


where *q*_1_ and *q*_2_ are respectively the displacement from the equilibrium position of oscillators^26^ (the dot over *q*_*j*_ (*j* = 1, 2) denotes time derivative), with *γ*_1_ and *γ*_2_ respectively their damping rates; *ω*_0_ = 2*π* × 32 GHz and *ω*_0_ + Δ are respectively resonance frequencies of the bright and dark modes (*γ*_2_ ≪ *γ*_1_ ≪ *ω*_0_); *g* is the parameter indicating the coupling strength of the bright mode with the incident radiation *E*; parameter *κ* denotes the coupling strength between the CW and SRR-pair. The last two terms on the left hand of Eq. (2) are provided by the hyperabrupt tuning varactors mounted onto gaps of the SRRs^18^,^19^,^20^. Thus the metamaterial structure suggested here is a coupled anharmonic oscillator system with the nonlinear coefficients *α* and *β* and driven by the electric field *E*. Note that when writing Eq. (1) and (2) we have taken a coordinate system in which the electric (magnetic) field *E* (*H*) is along *y* (*x*) direction and wavevector *k*_*f*_ is along *z* direction. We assume the frequency *ω*_*f*_ of the incident radiation is near *ω*_0_ (the natural frequency of the oscillator *q*_1_ in linear regime).

Evolution of the electric field is governed by the Maxwell equation


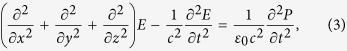


with the electric polarization intensity 
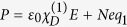
. Here *N* the density of unit cells, *e* is the unit charge, and 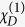 is the optical susceptibility of the background material (which is assumed to be linear). Note that the resonance of the two SRRs has nearly no contribution to the average field *E* since the asymmetric field distribution at the two SRRs cancels each other when PIT occurs^4^. In addition, since the wavelength of the incident field (8.5 mm) is much larger than the thickness of each unit cell (10 *μ*m), the electric field *E* seen by each meta-atom (i.e. the unit cell) is nearly homogeneous. Thus we can take an “electric-dipole approximation”, widely used in atomic physics and quantum optics^27^, to investigate the dynamics of the present system.

### PIT-ATS crossover

To treat them analytically, we assume


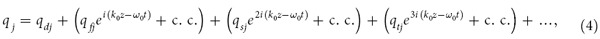



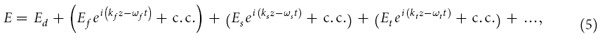


where *q*_*dj*_, *q*_*fj*_, *q*_*sj*_, *q*_*tj*_ (*j* = 1, 2) are respectively amplitudes of the direct-current, fundamental, second harmonic, third harmonic waves of the *j*th oscillator, with *k*_0_ (*ω*_0_) the wavenumber (frequency) of the fundamental wave; *E*_*d*_, *E*_*f*_, *E*_*s*_, *E*_*t*_ are respectively amplitudes of the direct-current, fundamental, second harmonic, third harmonic waves of the electric field, with *k*_*f*_ ≈ *k*_0_ (*ω*_*f*_), *k*_*s*_ (*ω*_*s*_), and *k*_*t*_ (*ω*_*t*_) respectively the wavenumber (frequency) of the fundamental, second harmonic, and third harmonic waves. Under rotating-wave and slowly varying envelope approximations, from Eqs (1) (2) and (3) we can obtain a series of equations for *q*_*μj*_ and *E*_*μ*_ (*μ* = *d*, *f*, *s*, *t*), which are presented in Methods.

We assume that the transverse spatial distribution of the radiation is large enough so that the diffraction effect (i.e. its dependence on *x* and *y*) of the system can be neglected. We use the standard method of multiple scales^28^,^29^ to derive the envelope equation of the radiation field based on the asymptotic expansion of *q*_*μj*_ and *E*_*μ*_ (see Methods). The first-order solution of the asymptotic expansion reads 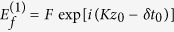, 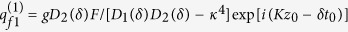, and 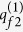 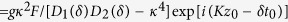 with 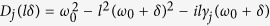 (*j*, *l* = 1, 2). Here *F* is a yet to be determined envelope function depending on the “slow” variables *z*_1_, *z*_2_ and *t*_1_, *δ* = *ω*_*f*_ − *ω*_0_ is frequency detuning, and *K* is the linear dispersion relation given by


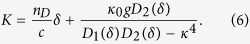


Shown in panels (d), (e), (f) of Fig. 2 is the absorption spectrum Im(*K*) (the imaginary part of *K*) as a function of frequency. When plotting the figure we used the damping rates *γ*_1_ ≈ 60 GHz and *γ*_2_ ≈ 10 GHz, which are nearly independent of *d*, whereas *κ* decreases from 152.5 GHz at *d* = 0.02 mm to 69 GHz at *d* = 0.38 mm. We see that the analytical result (the lower part of Fig. 2) fits well with the numerical one (the upper part of Fig. 2).

To obtain a clear physical explanation on the variation of the PIT transparency window for different *d* shown in Fig. 2, we make a detailed analysis on the linear dispersion relation. Near the resonance point (i.e. *δ* ≪ *ω*_0_), we have 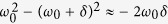, and hence


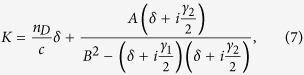


with *A* = *κ*_0_*g*/(2*ω*_0_) and *B* = *κ*^2^/(2*ω*_0_).

The linear dispersion relation (7) is similar to that obtained in Λ-type three-level atomic systems^1^. The PIT condition of the system reads *B*^2^ ≫ *γ*_1_*γ*_2_/4. To illustrate the interference effect between the bright and dark modes apparently, we make a spectrum decomposition on Im(*K*) using the technique developed recently in refs 30, 31, 32 for different coupling constant *κ*.

**Figure 3:**
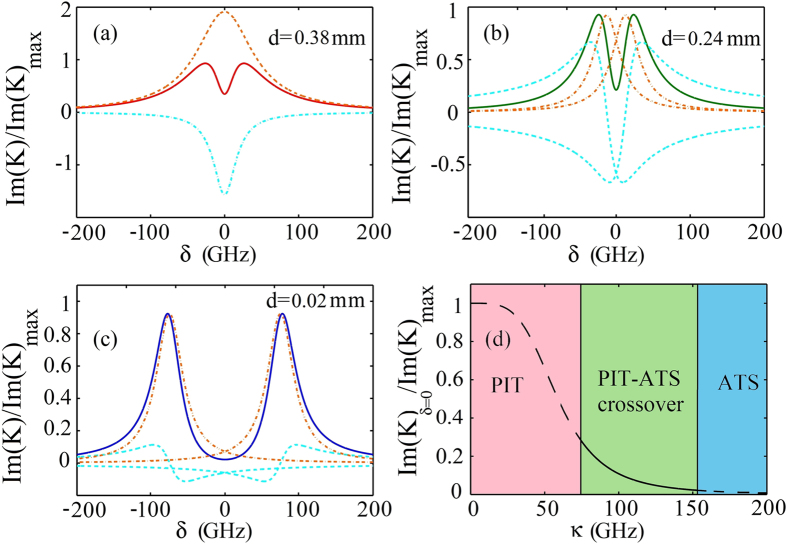
PIT, PIT-ATS crossover, and ATS in the plasmonic metamaterial. (**a**) The dashed (dotted-dashed) line is the result of the first (second) Lorentzian term in Eq. (8). The solid line is the sum of the two Lorentzian terms, giving the absorption spectrum Im(*K*) in the weak coupling (PIT) region (*d* = 0.38 mm). (**b**) The dashed-dotted lines denote the first two Lorentzian terms in Eq. (9); the dashed lines denote the third and fourth terms in Eq. (9). The solid line is the sum of the all four terms, giving Im(*K*) in the intermediate coupling (PIT-ATS crossover) region (*d* = 0.24 mm). (**c**). The same as (**b**) but with *d* = 0.02 mm, giving Im(*K*) in the strong coupling (ATS) region. (**d**) Transition from PIT to ATS when *κ* changes.

1. *Weak coupling region* (*κ*^2^ < (*γ*_1_ − *γ*_2_)*ω*_0_/2)): In this region, the absorption spectrum can be expressed as


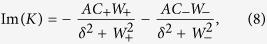


where 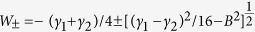 and 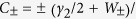 
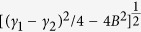
. We see that Im(*K*) consists of two Lorentzians terms. Figure 3(a) shows Im(*K*) as a function of *δ* for *κ* = 69 GHz. The dashed (dotted-dashed) line is the result of the first (second) Lorentzian term in Eq. (8), which is negative (positive). Because the two Lorentzians terms have the same center position but opposite sign, their superposition gives a destructive interference between CW and the SRR-pair. As a result, a small dip in Im(*K*) curve (i.e. the solid line) appears. Such phenomenon belongs PIT in nature.

2. *Intermediate coupling region* (*κ*^2^ > (*γ*_1_ − *γ*_2_)*ω*_0_/2)): In this case, we obtain


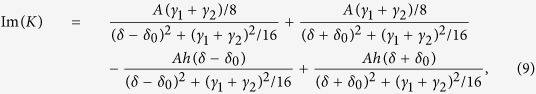


with 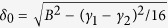 and *h* = (*γ*_2_ − *γ*_1_)/(8*δ*_0_). We see that the absorption spectrum is made of four terms. The first two terms are Lorentzians with the same width but different center position, which are two resonance peaks belonging respectively to the CW and the SRR-pair. The dip between the two Lorentzians can be interpreted as a gap between two resonances, which is a typical character of ATS. The next two terms are interference terms. Because they lowers the dip formed by the first two terms, a destructive interference (i.e. PIT) occurs. Since both PIT and ATS occur simultaneously in the system, we assign such phenomenon as *PIT-ATS crossover*. Plotted in Fig. 3(b) is the result of various terms in Eq. (9) and the total absorption spectrum Im(*K*) for *κ* = 82 GHz.

3. *Strong coupling region* (*κ*^2^ ≫ (*γ*_1_ − *γ*_2_)*ω*_0_/2)): The spectrum decomposition of Im(*K*) is still given by Eq. (9), but the destructive interference effect contributed by the last two terms plays a negligible role. Figure 3(c) shows the result for *κ* = 152.5 GHz. We see that in the strong coupling region the absorption spectrum of the electric field displays mainly the character of ATS.

In Fig. 3(d) we show the “phase diagram” of the system, where three regions (i.e. PIT, PIT-ATS crossover, and ATS) are indicated clearly. We see that a transition from PIT to ATS indeed occurs when *κ* changes from small to large values, which provides a satisfactory explanation for the phenomenon observed in our numerical simulation presented in the upper part of Fig. 2.

### Giant Kerr nonlinearity and low-power solitons

In order to explore the property of the nonlinear propagation of the radiation field, we go to high-order approximations. The solvability condition at the second order of the asymptotic expansion is *i*[∂*F*/∂*z*_1_ + (∂*K*/∂*ω*)∂*F*/∂*t*_1_] = 0, which means that the probe-pulse envelope *F* travels with the group velocity *V*_*g*_ = (∂*K*/∂*ω*)^−1^. The second-order solution is given in Methods.

With the above result we proceed to third order. The divergence-free condition in this order yields the nonlinear equation for the radiation field envelope *F*


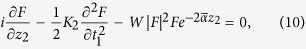


where 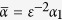, *α*_1_ 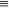 Im(*K*) is the coefficient describing linear absorption, *K*_2_ = ∂^2^*K*/∂*δ*^2^ is the coefficient describing group-velocity dispersion, and *W* is the coefficient describing Kerr effect. We have *W* = [*ω*_0_/(2*cn*_*D*_)]*χ*^(3)^, with


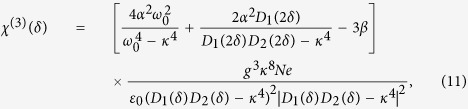


the third-order nonlinear susceptibility of the system.

**Figure 4:**
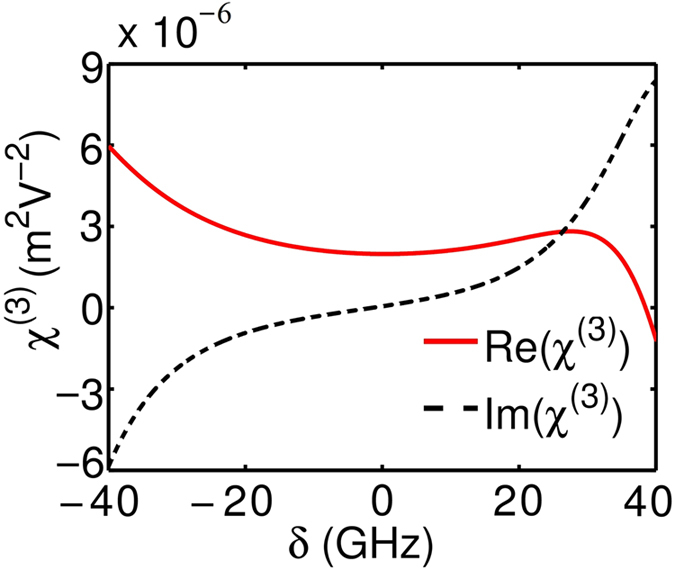
Giant Kerr nonlinearity in the PIT metamaterial. Illustrated are Re(*χ*^(3)^) (solid line) and Im(*χ*^(3)^) (dashed line) as functions of *δ*. System parameters used are given in the text.

Shown in Fig. 4 are curves of the real part of *χ*^(3)^, i.e., Re(*χ*^(3)^) (solid line), and its imaginary part, i.e., Im(*χ*^(3)^) (dashed line), as functions of *δ*. When plotting the figure, the system parameters used are *γ*_1_ = 60 GHz, *γ*_2_ = 10 GHz, *g* = 1.79 × 10^11^ C·kg^−1^, *κ*_0_ = 2.8 × 10^−8^ kg·C^−1^cm^−1^GHz^2^, and *κ* = 152.5 GHz, which are obtained by fitting the numerical result given in Figs 1 and 2. The parameters *α* = −6 × 10^14^ cm^−1^GHz^2^ and *β* = 1.18 × 10^23^ cm^−2^GHz^2^ are derived from the result given in ref. 18 (see Methods). From the figure we see that: (i) Re(*χ*^(3)^) has the order of magnitude 10^−6^ m^2^V^−2^. Thus the Kerr coefficient of the system 
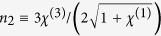
 (*χ*^(1)^ is the linear susceptibility) is of the order 10^−7^ m^2^V^−2^. (ii) Im(*χ*^(3)^), which contributes a nonlinear absorption to the radiation field, is much less than Re(*χ*^(3)^) when the system works in PIT transparency window (i.e., *δ* takes the values from −20 GHz to 20 GHz). Such suppression of the nonlinear absorption is also due to the PIT effect.

Although *χ*^(3)^ is originated from the hyperabrupt tuning varactors mounted onto the slits of the SRRs (i.e. *α* ≠ 0, *β *≠ 0) which can be large at GHz frequency, the giant enhancement of *χ*^(3)^ to the order of 10^−7^ m^2^V^−2^ obtained above is due to the contribution of the PIT effect. The physical reason is that the system we consider is a resonant one and works under the PIT condition, which can not only largely suppress the linear and nonlinearity absorptions of the radiation field, but also greatly strengthen the Kerr effect of the system, similar to the giant enhancement of the Kerr nonlinearity occurred in resonant atomic systems working under EIT condition^1^. Note that since Eqs (1) and (2) have quadratic nonlinearity resulting from the property of hyperabrupt tuning varactors, one is able to get a non-zero second-order nonlinear susceptibility *χ*^(2)^, which can also be enhanced via PIT effect. However, in the present work we do not discuss *χ*^(2)^ process (e.g. second harmonic generation) for which a phase matching condition is required, an interesting topic that will be considered elsewhere.

Based on the above results and returning to the original variables, we obtain the NLS equation


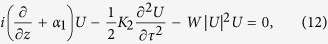


where *τ* = *t* − *z*/*V*_*g*_ and *U* = *ε F* exp(−*α*_1_*z*).

Generally, Eq. (12) has complex coefficients and hence it is a Ginzburg-Landau equation. However, due to the PIT effect the imaginary part of the complex coefficients can be made much smaller than their real part. Thus Eq. (12) can be converted into the dimensionless form


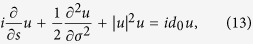


with *z* = −*L*_D_*s*, *τ* = *τ*_0_*σ*, *U* = *U*_0_*u*, and *d*_0_ = *L*_*D*_/*L*_*A*_. Here *τ*_0_, 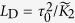, *L*_*A*_ = 1/*α*_1_, and 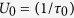 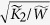 are typical pulse length, dispersion length, absorption length, and amplitude of the pulse, respectively (the tilde above the quantity means taking real part). Notice that for forming solitons *L*_*D*_ has been set to equal typical nonlinearity length 
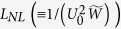
.

If *d*_0_ is small (this is the case we have here), the term *d*_0_*u* can be taken as a perturbation. One can use the perturbation method for solitons^33^ to solve the Eq. (13) to obtain a single-soliton solution under the perturbation. The result is given by


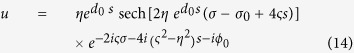


where *η*, *ς*, *σ*_0_ and *ϕ*_0_ are real free parameters determining the amplitude (as well as the width), propagating velocity, initial position, and initial phase of the soliton, respectively. When taking *η* = 1/2, *ς* = *σ*_0_ = *ϕ*_0_ = 0 and noting that *s* = −*z*/*L*_*D*_, we obtain the electric field corresponding the above single-soliton solution


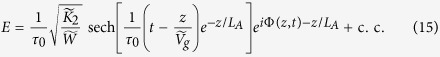


with 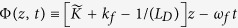, which describes a damped bright soliton traveling with velocity 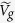.

For *δ* = 15 GHz and with other parameters given above, we obtain numerical values of the coefficients in Eq. (12), given by *α*_1_ = 0.0278 cm^−1^, *K*_2_ = (5.02 + 0.57*i*) × 10^−23^ cm^−1^s^2^, *W* = (4.62 + 0.72*i*) × 10^−2^ C^2^kg^−2^cm^−3^s^4^. We see that, as expected, the imaginary part of these coefficients are indeed much smaller than their real part. By taking *τ*_0_ = 1.5 × 10^−11^ s, we obtain *U*_0_ = 2.2 V cm^−1^, *L*_*D*_ = *L*_*NL*_ = 4.48 cm. In order to have such dispersion and nonlinearity lengthes, 20 layers of the unit cell array are needed to form the gigahertz soliton. With the above system parameters one has *L*_*A*_ = 36 cm, and hence *d*_0_ = 0.12, i.e. the dissipation in the system can be indeed taken as a perturbation. The physical reason of the long absorption length for the soliton propagation in the present system is also due to the PIT effect.

Note that the diffraction length of the system is given by 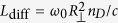 with *R*_┴_ the transverse spatial width of the radiation pulse. With the system parameters given above and if taking *R*_┴_ = 1.4 cm, we have *L*_diff_ = 43 cm, which is much larger than the dispersion length and nonlinearity length (4.48 cm). Thus in this case the diffraction effect of the system is indeed negligible.

The power associated with the soliton is given by Poynting’s vector integrated over the cross-section of the radiation in the transverse directions, i.e. *P* = ∫(**E** × **H**) · e_*z*_*dS*, where e_*z*_ is the unit vector in the propagation direction^29^. We obtain the peak power of the soliton 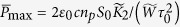, here 
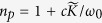
 is the refractive index and *S*_0_ is the area of the intensity distribution of the radiation field in the transverse directions. Using the above parameters and taking *S*_0_ = 6 × 10^−4^ m^2^, the peak power for generating the soliton is found to be 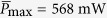, which means that to generate the soliton in present system a very low input power is needed. This is a drastically contrast to the case in conventional media such as optical fibers, where pico- or femto-second laser pulses are needed to reach a very high peak power (usually at the order of several hundred kW) to stimulate enough nonlinearity for the formation of solitons.

**Figure 5:**
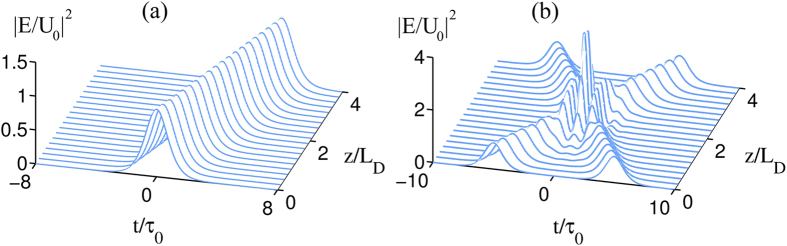
Propagation of the soliton and the interaction between two solitons. (**a**) The radiation intensity |*E*/*U*_0_|^2^ of the soliton as a function of *t*/*τ*_0_ and *z*/*L*_*D*_. (**b**) The collision between two solitons.

The stability of the gigahertz soliton is tested by using numerical simulation. Figure 5(a) shows the result of the radiation intensity |*E*/*U*_0_|^2^ of the soliton as a function of *t*/*τ*_0_ and *z*/*L*_*D*_. The solution is obtained by numerically solving Eq. (12) with the complex coefficients taken into account, with the initial condition given by *E*(0, *t*)/*U*_0_ = sech(*t*/*τ*_0_). We see that the shape of the soliton undergoes no apparent deformation during propagation.

The collision between two solitons is also studied numerically, with the result shown in Fig. 5(b). The initial condition is given by *E*(0, *t*)/*U*_0_ = sech[(*t* − 3.0)/*τ*_0_] + 1.2sech[1.2(*t* + 3.0)/*τ*_0_]. We see that the both solitons can resume their original shapes after the collision, indicating that solitons in the PIT-metamaterial are robust during interaction.

## Discussion

We have suggested a method for enhancing Kerr nonlinearity and realizing low-power gigahertz solitons via PIT in a new type of metamaterial, which can be constructed by an array of unit cell consisting of a cut-wire and a pair of varactor-loaded SRRs. We have demonstrated that the PIT in such system can not only mimic the electromagnetically induced transparency in coherent three-level atomic systems, but also exhibit a crossover from PIT to Autler-Townes splitting. We have further demonstrated that our system may possess a giant third-order nonlinear susceptibility and can be used to produce solitons with extremely low generation power.

The research results reported here raises the possibility for obtaining strong nonlinear effect of gigahertz radiation at very low intensity based on room-temperature metamaterials. The method presented in this work can be used to investigate other types of PIT systems, such as that proposed in ref. 6; furthermore, they can also be generalized to terahertz and even optical frequency regimes if related nonlinear elements are available.

## Methods

### Equations of motion for *q*_*μj*_ and *E*_*μ*_

Using making rotating-wave approximation, Eq. (1) and Eq. (2) become


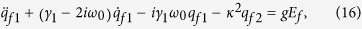



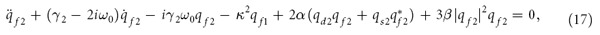



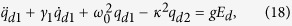



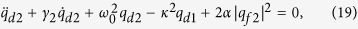



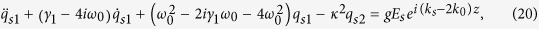



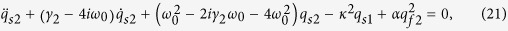



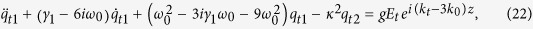



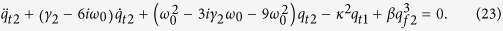


Under slowly varying envelope approximation, the Maxwell equation (3) is converted into


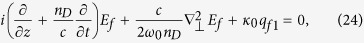



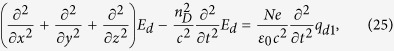



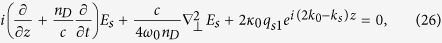



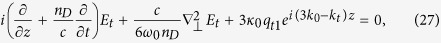


where 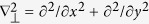, 
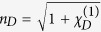
, and *κ*_0_ = (*Neω*_0_)/(2*ε*_0_*cn*_*D*_).

### Asymptotic expansion

We make the asymptotic expansion


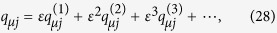



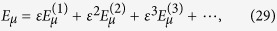


where *ε* is a dimensionless small parameter characterizing the amplitude of the incident electric field. All quantities on the right-hand side of the expansion are assumed as functions of the multi-scale variables *z*_*l*_ = *ε*^*l*^*z* (*l* = 0, 1, 2) and *t*_*l*_ = *ε*^*l*^*t* (*l* = 0, 1). Note that with the expansion based on the multiple-scale variables given here, a short-wavelength approximation is implied, i.e. the expansion is valid only for a propagation of a weak nonlinear pulse with finite *K* and finite *δ* (see Eq. (30) below). One can also consider other possibilities (e.g. long-wavelength approximation) that will result in different envelope equations, which will be discussed elsewhere.

Substituting the expansion (28) and (29) into the Eqs (16, 17, 18, 19, 20, 21, 22, 23, 24, 25, 26, 27) and comparing the coefficients of *ε*^*l*^ (*l* = 1, 2, 3, …), we obtain a chain of linear but inhomogeneous equations which can be solved order by order.

The first order (*l* = 1) solution is given by


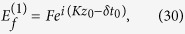



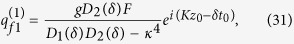



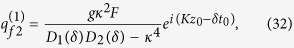


where *F* is a yet to be determined envelope function, and *K* is the linear dispersion relation given by Eq. (6).

At the second order (*l* = 2), we get the solution


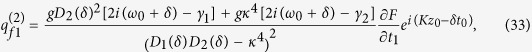



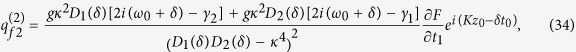



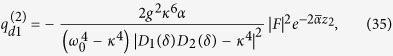



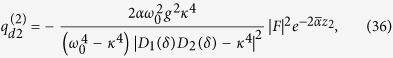



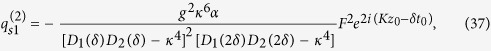



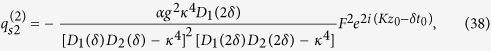


with 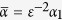, *α*_1_ 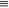 Im(*K*). A solvability (i.e. divergence-free) condition requires the envelope function *F* to satisfy the equation i[∂*F*/∂*z*_1_ + (1/*V*_*g*_)∂*F*/∂*t*_1_] = 0, with *V*_*g*_ = (∂*K*/∂*ω*)^−1^ being the group velocity of the envelope *F*.

At the third order (*l* = 3), a solvability condition results in the equation for *F*, i.e. Eq. (10).

### The calculation of nonlinear coefficients

The nonlinear property of the SRRs has been theoretically analyzed and experimentally measured in ref. 18. The value of *q* in ref. 18 represents the renormalized voltage and has the dimension of volt (V), while the value of *q*_2_ in our work represents the amplitudes of dark mode and has the dimension of centimeters (cm). To make a comparison we switch the dimension of our Eq. (1) and (2), which reads


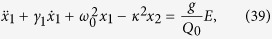



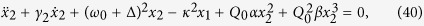


where *q*_*j*_ = *Q*_0_*x*_*j*_ (*j* = 1, 2). *x*_*j*_ has the dimension of V (voltage) and *Q*_0_ has the dimension cm·V^−1^. Thus, nonlinear coefficients *α* and *β* in our model (1) can be calculated by using the expressions of 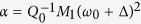 and 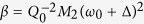, where *M*_1_ = −*M*/2(*V*_*p*_) and 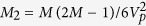 given in ref. 18.

## Additional Information

**How to cite this article**: Bai, Z. *et al.* Giant Kerr nonlinearity and low-power gigahertz solitons via plasmon-induced transparency. *Sci. Rep.* **5**, 13780; doi: 10.1038/srep13780 (2015).
